# Potential role of platelets for atherosclerotic events in rheumatoid arthritis

**DOI:** 10.1002/2211-5463.12531

**Published:** 2018-11-06

**Authors:** Rosa Vona, Manuela Di Franco, Lucrezia Gambardella, Anna C. Di Lollo, Cristina Iannuccelli, Guido Valesini, Walter Malorni, Elisabetta Straface

**Affiliations:** ^1^ Center for Gender‐Specific Medicine Biomarkers Unit Istituto Superiore di Sanità Rome Italy; ^2^ Department of Internal Medicine and Medical Specialities Rheumatology Unit Sapienza University of Rome Italy

**Keywords:** atherosclerosis, inflammation, oxidative stress, platelet activation, platelet aggregation, rheumatoid arthritis

## Abstract

Rheumatoid arthritis (RA) is a chronic inflammatory disease with increased risk of cardiovascular events and mortality that can be attributed to accelerated atherosclerosis. This pilot study aimed to investigate if changes in blood parameters were compatible with atherosclerotic events in RA patients. To this aim, 45 RA women (aged more than 18 years), and 25 age and gender‐matched healthy donors (HD) were included. Biomarkers of oxidative stress, platelet activation and platelet aggregation were analysed in RA patients at baseline and after six months of treatment with disease modifying anti‐rheumatic drugs (DMARDs). Flow cytometry analysis revealed that ca. 4% of platelets was in activated state (evaluated in term of Annexin V and PAC‐1 positivity) in RA patients at baseline, and that the 76% of platelets displayed mitochondrial hyperpolarization. Moreover, platelets from RA patients at baseline aggregated more than those from HD after whole blood treatment with adenosine diphosphate. Interestingly, platelet aggregation in patients at baseline positively correlated with disease activity measured by DAS28 score. After six months of treatment with DMARDs, platelet activation and platelet aggregation reached values comparable to those of HD. Our preliminary data suggest that platelets might play an active role in the atherosclerosis occurring in RA patients.

**Abbreviations**
ACD: acid‐citrate‐dextrose
ADP: adenosine diphosphate
DAS28: disease activity score
DMARDs: disease modifying anti‐rheumatic drugs
H_2_O_2_: hydrogen peroxide
HD: healthy donors
HMM: hyperpolarized mitochondria membrane
MMP: mitochondrial membrane potential
PRP: platelet‐rich plasma
PS: phosphatidylserine
RA: rheumatoid arthritis
ROS: reactive oxygen species

Rheumatoid arthritis (RA) is a chronic inflammatory disease with autoimmune pathogenesis and is associated with a significantly increased risk of cardiovascular mortality, mainly attributed to accelerated atherosclerosis 1. This latter is more prevalent in RA than in the general population, and atherosclerotic lesions progress at a faster rate and might be more prone to rupture leading to clinical events 2, 3. Although risks factors such as age, gender, smoking, hypertension or type‐2 diabetes have been evoked, the actors underlying the accelerated atherosclerosis in RA remain unclear. Atherosclerosis is a dynamic inflammatory process beginning with endothelial activation and followed by leukocyte recruitment, lipid oxidation and culminating with plaque destabilization and thrombosis 4, 5. In this context, endothelial dysfunction is a pivotal early step in atherosclerosis and it was defined as an abnormal response of the vascular wall to physiological stimuli 6. Platelets play a major role in the maintenance of endothelial integrity and homeostasis and represent an important linkage among inflammation, atherogenesis and thrombosis 7. They contain α‐granules rich in inflammatory cytokines, vasoactive substances, chemokines and stimulators aggregation, which are abundantly released upon platelet activation. Some of these agents propagate inflammation, increase vascular permeability and destroy cells at inflamed sites 8. Platelet activation can be enhanced by inflammatory mediators derived from leukocytes 9. Activated platelets exert a pro‐inflammatory action that can be largely ascribed to their ability to interact with monocytes 10, and take part in the switch from chronic inflammatory to pro‐thrombotic conditions due to their crosstalk with leukocytes, endothelium and coagulation system. Evidence for platelet activation in RA patients has been observed in both synovial fluid and blood samples 11. Compared with healthy individuals, increased levels of soluble platelet activation markers, CD40L and P‐selectin, are reported in patients with RA 12. Moreover, upon activation, platelets release pro‐inflammatory platelets microparticles (pMPs) whose levels are associated with disease activity in RA 13.

The major goal of this work was to evaluate if changes in platelet (dys)function reflect increased risk of atherosclerosis in patients with RA. To this aim, a pilot study in peripheral blood from 45 RA patients at baseline and after six months of treatment with disease modifying anti‐rheumatic drugs (DMARDs) was performed. Specifically, molecules involved in platelet activation and platelet aggregation were analysed before and after therapy with DMARDs.

## Results and Discussion

In the pilot study, 45 women with RA (median age 44 years) and 25 age‐ and gender‐matched healthy donors (HD) were included. RA patients were clustered into two study groups. The former included 23 patients with a mean disease duration of 24 weeks (range 5–48) and high‐moderate disease activity (disease activity score 28, DAS28 4.7 ± 1.0). The latter integrated 22 RA patients characterized by a mean disease duration of 144 weeks (range 68–324) and DAS28 value of 4.3 ± 10.8. Patients at baseline are named RA‐T0. The groups were treated, respectively, with Methotrexate and anti‐TNF‐alfa as disease modifying anti‐rheumatic drugs (DMARDs) and monitored in a 6 months long survey. Patients after the treatment are referred as RA‐F.U. (RA‐follow‐up).

**Figure 1:**
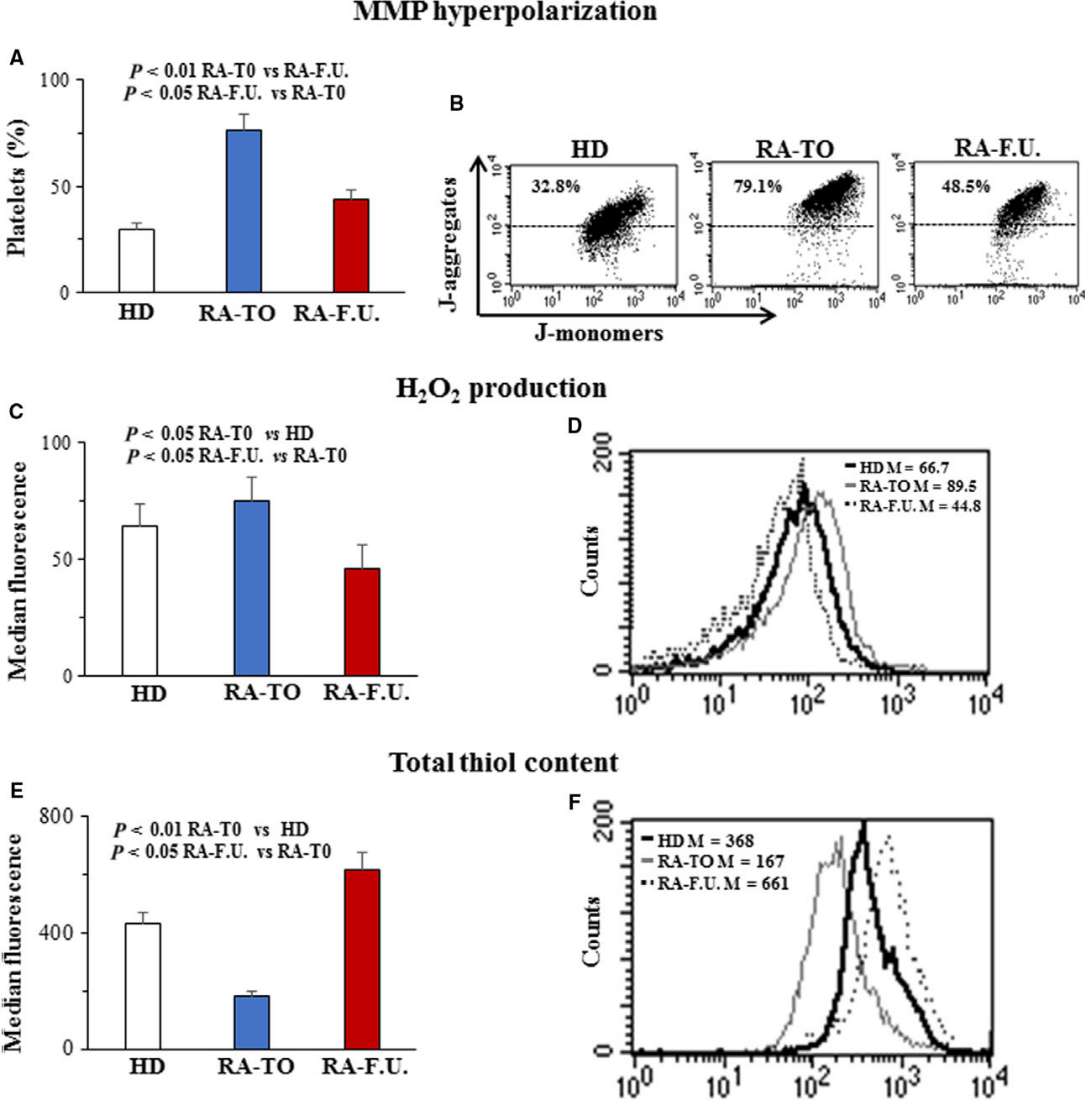
Oxidative stress and mitochondria in platelets from RA patients. Flow cytometry evaluation of: (A) Percentage of platelets with hyperpolarized mitochondria membrane (HMM); (C) Levels of hydrogen peroxide (H_2_O_2_); and (E) Levels of total thiol content. The numbers refers to mean ± SD of 45 women with RA, baseline (RA‐T0) and six months after DMARDs treatment (RA‐F.U.), and 25 age‐ and gender‐matched healthy donors (HD). In B, typical flow cytometric measure of HMM in platelets from HD (left panel), RA‐T0 (middle) and RA‐F.U typical subjects. In D, typical flow cytometric measure of H_2_O_2_ levels in platelets from HD and RA (RA‐T0 and RA‐F.U.) representative patients. In F, typical flow cytometric measure of total thiol content in platelets from a representative HD and a representative RA patient (RA‐T0 and RA‐F.U.). For each patient, flow cytometry analysis was conducted in triplicate. Bar graph showing the results obtained in three independent experiments and reported as mean ± SD of the median fluorescence intensity. The Student's *t* test was performed.

In RA‐F.U., disease activity was significantly reduced in both Methotrexate‐ (DAS28 = 3.2 ± 1.0) and anti‐TNF‐alfa‐treated (DAS28 = 2.9 ± 0.5) groups. RA systemic inflammation is associated with endothelial damage and accelerated atherosclerosis 14, 15. By using flow cytometry, the mitochondrial membrane potential (MMP) in platelets from both RA‐T0 and RA‐F.U. patients was measured. As shown in Fig. 1A, RA‐T0 patients showed a significantly (*P* < 0.01) higher percentage of platelets (76%) with hyperpolarized mitochondria membrane (HMM) as compared to healthy donors (30%). Interestingly, in treated patients the percentage of platelets with HMM decreased significantly (*P* < 0.05) to values comparable to those measured in HD (44% vs 30%). Typical flow cytometric measure of HMM in platelets from representative HD (left panel), RA‐T0 (middle) and RA‐F.U. (right) are in Fig. 1B. During endothelial dysfunction or under pathological conditions, reactive oxygen species (ROS) production increases and the platelets respond with specific biochemical and morphological changes. In this context, mitochondria play a key role, being able to both generate ROS driving redox‐sensitive events, and respond to ROS‐ mediated changes of the cellular redox state 16. Given that HMM involves an increased production of ROS, the levels of hydrogen peroxide (H_2_O_2_) in platelets were measured by using flow cytometry. As shown in Fig. 1C, the levels of H_2_O_2_ measured in platelets from RA‐T0 were significantly (*P* < 0.05) higher with respect to those measured in HD. At the follow‐up, H_2_O_2_ levels dropped significantly (*P* < 0.05) and reached values even below those measured in HD. Typical flow cytometric measure of H_2_O_2_ levels in platelets from representative HD and RA patients (RA‐T0 and RA‐F.U.) are shown in Fig. 1D. In order to assess whether H_2_O_2_ production could lead to redox alterations within the platelets, total thiol levels were measured. As shown in Fig. 1E the total thiol content decreased significantly (*P* < 0.01) in platelets from RA‐T0 patients with respect to those from HD. Interestingly, in RA‐F.U. the total thiol content increased significantly (*P* < 0.05) with respect to those measured in platelets from RA‐T0 patients and reached values higher than those measured in platelets from HD. Typical flow cytometric measure of total thiol content in platelets from a representative HD and a representative RA patient (RA‐T0 and RA‐F.U.) are in Fig. 1F.

**Figure 2:**
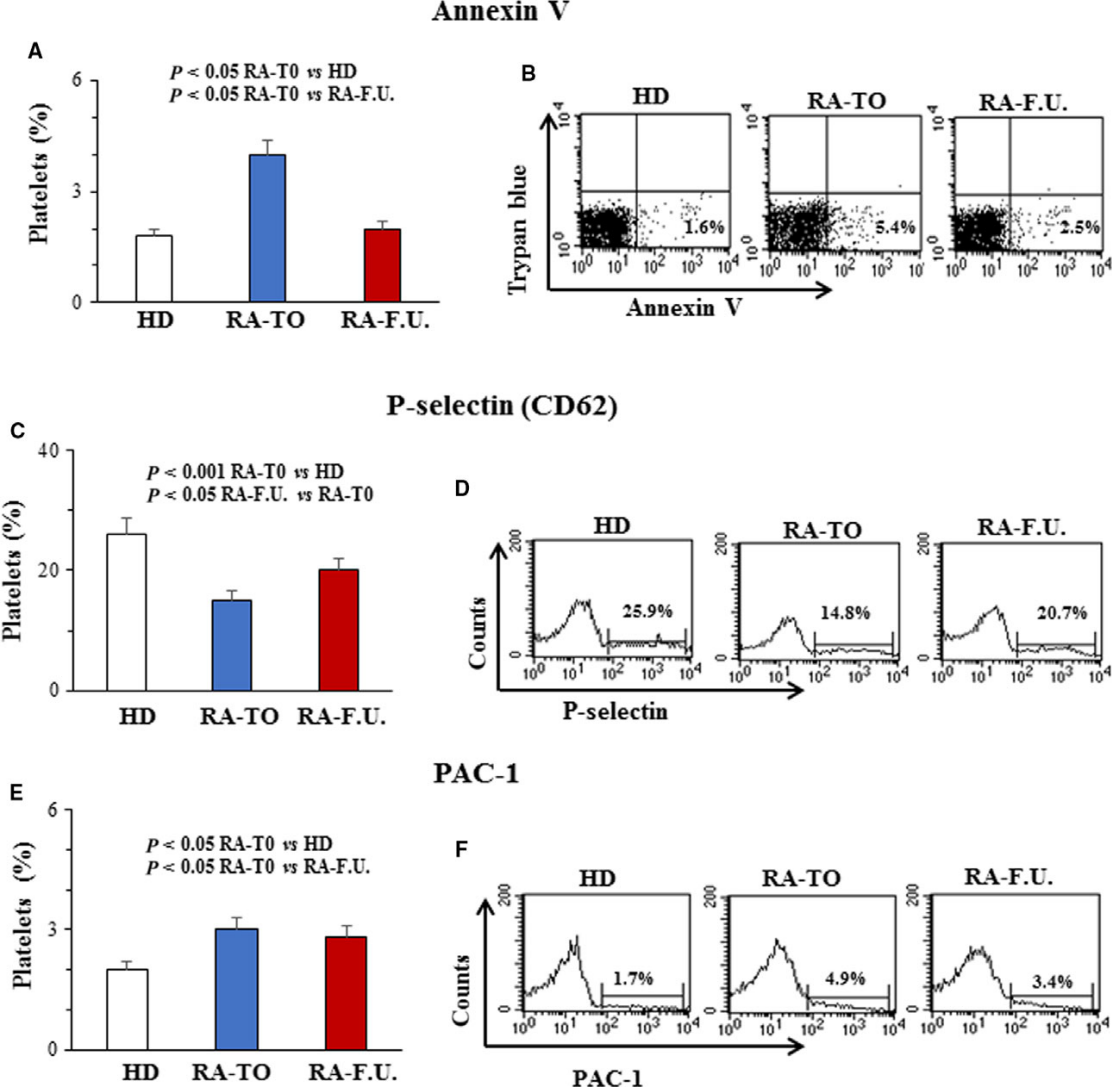
Molecules involved in platelet activation. Evaluation by flow cytometry of platelet positives to: (A) Annexin V; (C) P‐selectin (CD62) and (E) PAC‐1. The numbers are the mean ± SD of 45 women with RA (RA‐T0 and RA‐F.U.) and 25 age‐ and gender‐matched healthy donors (HD). The Student's *t* test was performed. For each patient, flow cytometry analysis was conducted in triplicate. For each molecule, analysis of platelets from a representative HD and a representative RA patient (RA‐T0 and RA‐F.U.) are shown in B, D and F).

**Figure 3:**
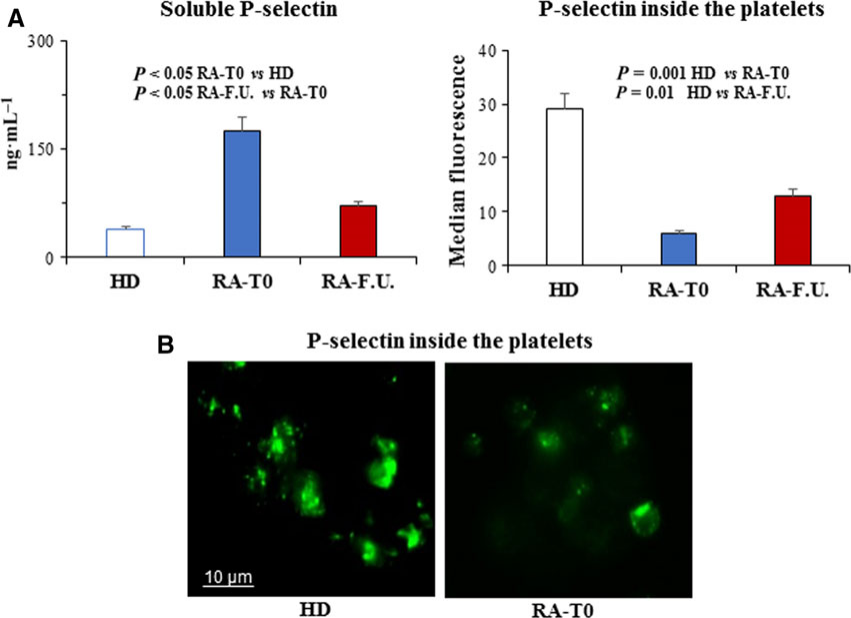
Platelet shedding and aggregation. (A, left panel) Spectrophotometric analysis of soluble P‐selectin in the plasma from HD and RA patients (RA‐T0 and RA‐F.U.). (A, right panel) Flow cytometry analysis of P‐selectin inside the platelets from HD and RA patients (RA‐T0 and RA‐F.U.). The numbers are the mean ± SD of 45 women with RA (RA‐T0 and RA‐F.U.) and 25 age‐ and gender‐matched healthy donors (HD). For each patient, flow cytometry analysis was conducted in triplicate. The Student's *t* test was performed. (B) Two representative images obtained by immunofluorescence microscopy showing content and distribution of P‐selectin in platelets from a HD (left panel) and a RA patient (right panel).

**Figure 4:**
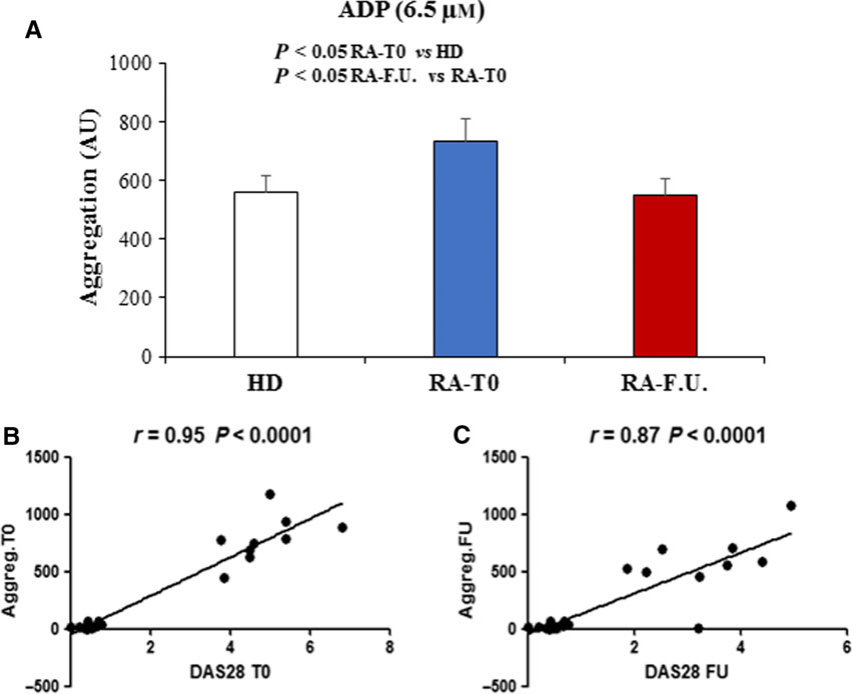
Platelet aggregation and DAS28 correlation. (A) Platelet aggregation evaluated in the whole blood from HD and RA patients (RA‐T0 and RA‐F.U.). The measurement was carried out after addition of 6.5 μMol ADP by an impedance multiplate aggregometer. The values are expressed in arbitrary aggregation units (AU). For each patient, platelet aggregation was conducted in triplicate. Bar graph showing the results obtained and reported as mean ± SD. The Student's t test was performed. (B) Correlation evaluated in early RA patients at baseline (RA‐T0) (left panel) and in early RA patients at F.U. (right panel). Pearson's correlation analysis was performed.

In order to determine platelet activation by mitochondrial hyperpolarization and H_2_O_2_ generation, phosphatidylserine (PS) externalization, surface P‐selectin and activation of integrin GP αIIbβ3 were measured by flow cytometry. PS is one of the four major phospholipids distributed symmetrically in the bilayer of cell plasma membrane and is normally confined to the membranes inner leaflet. When exposed on the outer membrane surface of activated platelets, PS mediates platelet pro‐coagulant function and regulates platelet life span 17. PS causes coagulation and thrombosis by providing a suitable surface for assembly of the pro‐thrombinase complex, which converts prothrombin to thrombin 18. P‐selectin is stored in α‐granules in the inner of platelets and it is expressed on the platelet surface after activation. It is also important in platelet–leukocyte interaction 19. In fact, through P‐selectin, platelets bind to the P‐selectin glycoprotein ligand 1(PSGL‐1 or CD162) on leukocytes, and multicellular aggregates, which promote the release of chemokines and cytokines further activating leukocytes and promoting atherosclerosis. Platelet P‐selectin is also required for the initial ‘rolling’ of activated platelets on accelerating atherosclerosis atherosclerotic arteries 20. Moreover, it has been shown that circulating de‐granulated platelets rapidly lose their surface P‐selectin into the plasma pool 21. Elevated level of soluble P‐selectin (sP‐selectin) has been suggested to represent not only a biomarker of inflammation but, also, an active player in the process. It has been suggested that this could enhance pro‐coagulant leukocyte microparticle generation and integrin activation accelerating atherosclerosis 22, 23. Integrin αIIbβ3 is platelet fibrinogen receptor that plays a vital role in platelet thrombus formation because it is required for platelet–platelet interactions 24. Integrin αIIbβ3 may also contribute to primary platelet adhesion to the exposed sub‐endothelial matrices and possibly also to activated endothelial cells 25. Few studies highlighted the role of platelets in RA and particularly in the early phase of disease. As shown in Fig. 2A, in RA‐T0 patients the percentage of platelets with PS externalization (Annexin V positives) was significantly (*P* < 0.05) higher than that measured in HD. At the follow‐up, this percentage significantly (*P* < 0.05) decreased and reached values similar to those measured in HD. In Fig. 2B dot plots showing platelets from HD (left panel), RA‐T0 (middle) and RA‐F.U. (right) representative patients are shown. The numbers represent the percentage of Annexin V single positive cells (bottom right quadrant). Conversely, in RA‐T0 patients the percentage of platelets with P‐selectin at the cell surface was significantly lower in comparison to that detected in HD (*P* < 0.001, Fig. 2C). In RA‐F.U., the percentage of platelets with P‐selectin at the cell surface increased and reached values similar to those measured in HD. Typical flow cytometric measures of P‐selectin, in platelets from HD (left panel), RA‐T0 (middle) and RA‐F.U. (right) representative patients are shown in Fig. 2D. As for GP αIIbβ3 activation (evaluated in term of PAC‐1 positivity) we found that in RA‐T0 patients the percentage of platelets positive to PAC‐1 was significantly (*P* < 0.05) higher with respect to that detected in HD (Fig. 2E). This population of platelets was also high at the follow‐up. Typical flow cytometric measures of PAC‐1 in platelets from HD (left panel), RA‐T0 (middle) and RA‐F.U. representative patients (right) are shown in Fig. 2F. The plasmatic levels of sP‐selectin were measured by using an immunoassay kit. As shown in Fig. 3A (left panel), we found that the levels of sP‐selectin were significantly higher (*P* < 0.05) in RA‐T0 patients than those measured in both HD and RA‐F.U. Interestingly, high levels of sP‐selectin associated were with decreased contents of P‐selectin inside the platelet of RA patients. Specifically, the P‐selectin content in platelets from RA‐T0 patients was significantly (*P* = 0.001) lower than that measured in platelets from HD and raised at comparable levels to the healthy controls after DMARDs treatments (Fig. 3A, right panel). We further investigated P‐selectin distribution inside platelets by using immunofluorescence analysis. In Fig. 3B two representative images obtained by immunofluorescence microscopy showing P‐selectin content in platelets from HD (left panel) and RA (right) patient are depicted. Platelet–platelet and platelet–leukocyte adhesion is crucial in atherosclerosis and thrombus formation. As a consequence, the platelet aggregation was measured in the whole blood from RA patients and HD after the addition of adenosine diphosphate (ADP; 6.5 μm) and collagen (3.2 μg·mL^−1^). ADP is a platelet physiological agonist released from erythrocytes and platelets that, under physiological conditions, triggers platelet clotting via the P2Y12 7, 26. Collagen is also a platelet agonist that, under physiological conditions, triggers platelet aggregation via the glycoprotein (GP) VI transmembrane receptor. As shown in Fig. 4A, platelets from RA‐T0 patients aggregated significantly (*P* < 0.05) more than those from HD and RA‐F.U. patients after ADP addition. Interestingly, a very strong (*r* = 0.95, *P* < 0.001) and strong (*r* = 0.87, *P* < 0.001) correlations between platelet aggregation and active disease (DAS28) was scored in RA‐T0 and RA‐F.U. samples, respectively (Fig. 4B,C).

Overall, these data confirm the key role of the platelet aggregation in RA and may explain the higher incidence of atherosclerosis in RA affected patients than in general population. Conversely, after collagen addition no differences were detected in aggregation of platelets from patients at RA‐T0 and RA‐F.U. respect to that in HD (539 vs 517 = values expressed in arbitrary aggregation units) (data no shown). Although the sample size was small, the two patient cohorts (patients treated with Methotrexate and patients treated with anti‐TNF‐alfa) were analysed separately. No significant differences were detected at baseline. Conversely, biologic therapy exerted a greater effect on platelet aggregation induced by ADP agonist than that observed for Methotrexate. In particular, ADP‐induced platelet aggregation was reduced by 19% in patients treated with Methotrexate and by 27% in patients treated with the biologic therapy. No differences were detected in the percentage of platelets expressing surface integrin αIIbβ3 (evaluated in term of PAC‐1 positivity) and phosphatidylserine (evaluated in term of Annexin V positivity) upon DMARDs treatments (see Fig. S1).

On the basis of these data, it can be hypothesized that biologic therapy acts mainly on the aggregation of circulating blood cells, which in turn lead to the activation of leukocytes and promoting atherosclerosis mediated by chemokines and cytokines release.

In conclusion, during RA endothelial dysfunction, the platelets respond with specific biochemical and morphological changes, including increased of HMM, ROS production and PS externalization, which mediate platelet pro‐coagulant functions. Moreover, our results indicate that biologic therapy reduces the amounts of platelets expressing surface P‐selectin and the capacity of platelets to aggregate after stimulation with ADP. Our data had shown a very strong correlation between platelet aggregation and active disease, suggesting that platelets may play a key role in the atherosclerosis occurring in RA patients. Finally, further studies on larger patient cohorts are required to verify such hypothesis.

## Experimental procedures

### Study design

In our study, 45 women (aged more than 18 years) with RA, diagnosed according to 2010 ACR (criteria from Early Athritis Clinic of Rheumatologia), and 25 healthy subjects (HD) age matched, were recruited at the Department of Internal Medicine and Medical Specialities of Sapienza University of Rome. The study was reviewed and approved by the Local Ethical Committee at the Sapienza University, and a written informed consent was obtained from all patients. All clinical investigations have been conducted according to the principles expressed in the Declaration of Helsinki. Complete clinical examination and clinometric assessment by DAS28 (a composite measure of RA inflammatory disease activity) 27 have been performed in all patients. We considered exclusion criteria: smoking, hypercholesterolaemia, arterial hypertension, presence of cardiovascular diseases, type 1 and 2 diabetes, cancer, use of platelet aggregation inhibitors, anticoagulant drugs and all anti‐inflammatory non‐steroidal drugs. The study was conducted in 2 years and patients were assessed at baseline and after 6 months follow‐up.

### Platelet isolation

Fresh whole blood samples were collected in acid‐citrate‐dextrose tubes (ACD; NIH formula A), and immediately centrifuged at 200 g for 12 min at room temperature to separate platelet‐rich plasma (PRP). Additional ACD was added (one part ACD per three parts PRP) and platelets were pelleted at 800 g for 15 min as previously reported 28.

### Plasma isolation

For plasma isolation, blood was centrifuged at 3000 ***g*** for 10 min at room temperature. Plasma was removed, aliquoted and frozen until analyses.

### Mitochondrial membrane potential

The MMP of platelets was studied using 5‐5¢,6‐6¢‐tetrachloro‐1,1¢,3,3¢ tetraethylbenzimidazol‐ carbocyanine iodide (JC‐1; catalogue number: T‐3168, Molecular Probes, Eugene, OR, USA) as previously described 29.

### Evaluation of the redox state

To evaluate H_2_O_2_ production, platelets were incubated with 10 μmol·L^−1^ dihydrorhodamine 123 (DHR 123, Molecular Probes) for 15 min at 37 °C 30. Intracellular content of total thiols was explored by using 5‐chloromethylfluoresceindiacetate (CMFDA; Molecular Probes). Samples were then analysed using a fluorescence‐activated cell‐sorting (FACS) flow cytometer (Becton Dickinson, Mountain View, CA, USA). The median values of fluorescence intensity histograms were used to provide semi‐quantitative evaluation of reduced thiol content and H_2_O_2_ production.

### Platelet activation

Annexin V positive platelets were detected by using fluorescein isothiocyanate‐ conjugated Annexin V, which labels platelets with phosphatidylserine externalization.

For P‐selectin (CD62) and PAC‐1 detection samples were stained at 4 °C for 30 min with monoclonal anti‐CD62 IgG‐Phycoerythrin‐conjugated and monoclonal anti PAC‐1 IgG‐FITC‐conjugated (Becton Dickinson), respectively.

### Detection of P‐selectin inside the platelets

For P‐selectin detection, samples were fixed with 4% paraformaldehyde, permeabilized with 0.5% Triton X‐100 (Sigma, Milan, Italy) and stained with a specific monoclonal antibody IgG FITC‐conjugated (Chemicon International, Inc., Temecula, CA, USA) 31. Finally, all samples were analysed with an OLYMPUS BX51 fluorescence micro‐ scope equipped with a CCD camera (Carl Zeiss, Jena, Germany). Alternatively, platelets were also analysed on a FACScan flow cytometer (Becton Dickinson) for quantitative analyses.

### Detection of soluble P‐selectin

sP‐selectin was measured in the plasma by using the Human sP‐selectin (R&D Systems, Minneapolis, MN, USA) commercial immunoassay Kit.

### Platelet aggregation

Platelet aggregation was evaluated in the whole blood from HD and RA patients. The measurement was carried out after the addition of 6.5 μMol ADP (Dynabyte, Munich, Germany) or 3.2 μg·mL^−1^ collagen (Dynabyte) by using an impedance multiplate aggregometer.

### Statistical analyses

Cytofluorimetric results were statistically analysed by using the parametric Kolmogorov–Smirnov test using cell quest Software (San Jose, CA, USA). A least 20 000 events were acquired. The median values of fluorescence intensity histograms were used to provide a semi‐quantitative analysis. Results are displayed as average value ± standard deviation, unless otherwise specified. Statistical analysis was performed with the statistical package Prism 6 (graphpad Software, La Jolla, CA, USA). The Student's *t* test and the Pearson's correlation analysis were performed. Statistical significance was set at *P* < 0.05.

## Author contributions

MD, RV and ES designed the study. RV performed platelet aggregation. LG performed static and flow cytometry. ACD and CI recruited patients. GV and WM revised the manuscript.

## Conflict of interest

The authors declare no conflict of interest.

## Supporting information


**Fig. S1.** Analysis of data from two rheumatoid arthritis (RA) patient cohorts: RA patients treated with Methotrexate and RA patients treated with anti‐TNF‐alfa.

## References

[1] Smolen JS , Aletaha D and McInnes IB (2016) Rheumatoid arthritis. Lancet 388, 2023–2038. 27156434 10.1016/S0140-6736(16)30173-8

[2] Hannawi S , Haluska B , Marwick TH and Thomas R (2016) Atherosclerotic disease is increased in recent onset rheumatoid arthritis: a critical role for inflammation. Arthritis Res Ther 9, R116. 10.1186/ar2323 PMC2246234 17986352

[3] Mahmoudi M , Aslani S , Fadaei R and Jamshidi AR (2017) New insights to the mechanisms underlying atherosclerosis in rheumatoid arthritis. Int J Rheum Dis 20, 287–297. 28205331 10.1111/1756-185X.12999

[4] Skeoch S and Bruce IN (2015) Atherosclerosis in rheumatoid arthritis: is it all about inflammation? Nat Rev Rheumatol 11, 390–400. 25825281 10.1038/nrrheum.2015.40

[5] Libby P (2008) Role of inflammation in atherosclerosis associated with rheumatoid arthritis. Am J Med 12, S21–S31. 10.1016/j.amjmed.2008.06.014 18926166

[6] Kinlay S , Libby P and Ganz P (2001) Endothelial function and coronary artery disease. Curr Opin Lipidol 12, 383–389. 11507322 10.1097/00041433-200108000-00003

[7] Davi G and Patrono C (2007) Platelet activation and atherothrombosis. N Engl J Med 357, 2482–2494. 18077812 10.1056/NEJMra071014

[8] Smyth SS , McEver RP , Weyrich AS , Morrell CN , Hoffman MR , Arepally GM , French PA , Dauerman HL and Becker RC (2009) Platelet functions beyond hemostasis. J Thromb Haemost 7, 1759–1766. 19691483 10.1111/j.1538-7836.2009.03586.x

[9] Rondina MT , Weyrich AS and Zimmermann GA (2013) Platelets as cellular effectors of inflammation in vascular diseases. Circ Res 112, 1506–1519. 23704217 10.1161/CIRCRESAHA.113.300512 PMC3738064

[10] Rong MY , Wang CH , Wu ZB , Zeng W , Zheng ZH , Han Q , Jia JF , Li XY and Zhu P (2014) Platelets induce a pro‐inflammatory phenotype in monocytes via the CD147 pathway in rheumatoid arthritis. Arthritis Res Ther 16, 478. 25404518 10.1186/s13075-014-0478-0 PMC4298113

[11] Boilard E , Blanco P and Nigrovic PA (2012) Platelets: active players in the pathogenesis of arthritis and SLE. Nat Rev Rheumatol 8, 534–542. 22868927 10.1038/nrrheum.2012.118

[12] Pamuk GE , Vural O , Turgut B , Demir M , Pamuk ON and Cakir N (2008) Increased platelet activation markers in rheumatoid arthritis: are they related with subclinical atherosclerosis? Platelets 19, 146–154. 17852775 10.1080/09537100701210057

[13] Knijff‐Dutmer EA , Koerts J , Nieuwland R , Kalsbeek‐Batenburg EM and van de Laar MA (2002) Elevated levels of platelet microparticles are associated with disease activity in rheumatoid arthritis. Arthritis Rheum 46, 1498–1503. 12115179 10.1002/art.10312

[14] Di Franco M , Spinelli FR , Metere A , Gerardi MC , Conti V , Boccalini FIannuccelli C , Ciciarello F , Agati L and Valesini G (2012) Serum levels of asymmetric dimethylarginine and apelin as potential markers of vascular endothelial dysfunction in early rheumatoid arthritis. Mediators Inflamm 2012, 347268. 10.1155/2012/347268 PMC3420101 22927708

[15] Totani L and Evangelista V (2010) Platelet‐leukocyte interactions in cardiovascular disease and beyond. Arterioscler Thromb Vasc Biol 30, 2357–2361. 21071701 10.1161/ATVBAHA.110.207480 PMC3076621

[16] Pietraforte D , Vona R , Marchesi A , Tarissi de Jacobis I , Villani A , Del Principe D and Straface E (2014) Redox control of platelet functions in physiology and pathophysiology. Antioxid Redox Signal 2, 177–1793. 10.1089/ars.2013.5532 24597688

[17] Choo HJ , Kholmukhamedov A , Zhou C and Jobe S (2017) Inner mitochondrial membrane disruption links apoptotic and agonist‐initiated phosphatidylserine externalization in platelets. Arterioscler Thromb Vasc Biol 37, 1503–1512. 28663253 10.1161/ATVBAHA.117.309473 PMC5560492

[18] Stevic I , Chan HH , Berry LR , Chander A and Chan AK (2013) Inhibition of the prothrombinase complex on red blood cells by heparin and covalent antithrombin‐heparin complex. J Biochem 153, 103–110. 23100269 10.1093/jb/mvs129 PMC3528004

[19] Bernardo A , Ball C , Nolasco L , Choi H , Moake JL and Dong JF (2005) Platelets adhered to endothelial cell‐bound ultra‐large von Willebrand factor strings support leukocyte tethering and rolling under high shear stress. J Thromb Haemost 3, 562–570. 15748247 10.1111/j.1538-7836.2005.01122.x

[20] Michelson AD , Barnar MR , Hechtman HB , MacGregor H , Connolly RJ , Loscalzo J and Valeri CR (1996) In vivo tracking of platelets: circulating degranulated platelets rapidly lose surface Pselectin but continue to circulate and function. Proc Natl Acad Sci USA 93, 11877–11882. 8876231 10.1073/pnas.93.21.11877 PMC38152

[21] Panicker SR , Mehta‐D'souza P , Zhang N , Klopocki AG , Shao B and McEver RP (2017) Circulating soluble P‐selectin must dimerize to promote inflammation and coagulation in mice. Blood 130, 181–191. 28515093 10.1182/blood-2017-02-770479 PMC5510792

[22] Hope SA and Meredith IT (2003) Cellular adhesion molecules and cardiovascular disease. Part II. Their association with conventional and emerging risk factors, acute coronary events and cardiovascular risk prediction. Intern Med J 33, 450–462. 14511199 10.1046/j.1445-5994.2003.00379.x

[23] Canobbio I , Balduini C and Torti M (2004) Signalling through the platelet glycoprotein Ib‐V‐IX complex. Cell Signal 16, 1329–1344. 15381249 10.1016/j.cellsig.2004.05.008

[24] Bennett JS (2015) Regulation of integrins in platelets. Biopolymers 104, 323–333. 26010651 10.1002/bip.22679

[25] Zharikov S and Shiva S (2013) Platelet mitochondrial function: from regulation of thrombosis to biomarker of disease. Biochem Soc Trans 41, 1118–1123. 10.1042/BST20120327 23356269

[26] Rivera J , Lozano ML , Navarro‐Núñez L and Vicente V (2009) Platelet receptors and signaling in the dynamics of thrombus formation. Haematologica 94, 700–711. 19286885 10.3324/haematol.2008.003178 PMC2675683

[27] Prevoo ML , van‘t Hof MA , Kuper HH , van Leeuwen MA , van de Putte LB and van Riel PL (1995) Modified disease activity scores that include twenty‐eight‐joint counts. Development and validation in a prospective longitudinal study of patients with rheumatoid arthritis. Arthritis Rheum 38, 44–48. 7818570 10.1002/art.1780380107

[28] Shcherbina A and Remold‐O'Donnell E (1999) Role of caspase in a subset of human platelet activation responses. Blood 93, 4222–4231. 10361119

[29] Matarrese P , Gambardella L , Cassone A , Vella S , Cauda R and Malorni W (2003) Mitochondrial membrane hyperpolarization hijacks activated T lymphocytes toward the apoptotic‐prone phenotype: homeostatic mechanisms of HIV protease inhibitors. J Immunol 170, 6006–6015. 12794128 10.4049/jimmunol.170.12.6006

[30] Frey T (1997) Correlated flow cytometric analysis of terminal events in apoptosis reveals the absence of some changes in some model systems. Cytometry 28, 253–263. 9222111 10.1002/(sici)1097-0320(19970701)28:3<253::aid-cyto10>3.0.co;2-o

[31] Straface E , Gambardella L , Metere A , Marchesi A , Palumbo G , Cortis E , Villani A , Pietraforte D , Viora M , Malorni W *et al* (2010) Oxidative stress and defective platelet apoptosis in naïve patients with Kawasaki disease. Biochem Biophys Res Commun 392, 426–430. 20079717 10.1016/j.bbrc.2010.01.040

