# Supplementary material for: Potential role of platelets for atherosclerotic events in rheumatoid arthritis
Source: FEBS Open Bio. 2018 Nov 6;8(12):1888–96. doi: 10.1002/2211-5463.12531 (PMC6275263; doi:10.1002/2211-5463.12531)
Supplement: Supplementary file 1 — Fig. S1. Analysis of data from two rheumatoid arthritis (RA) patient cohorts: RA patients treated with Methotrexate and RA patients treated with anti‐TNF‐alfa. [file FEB4-8-1888-s001.pdf]

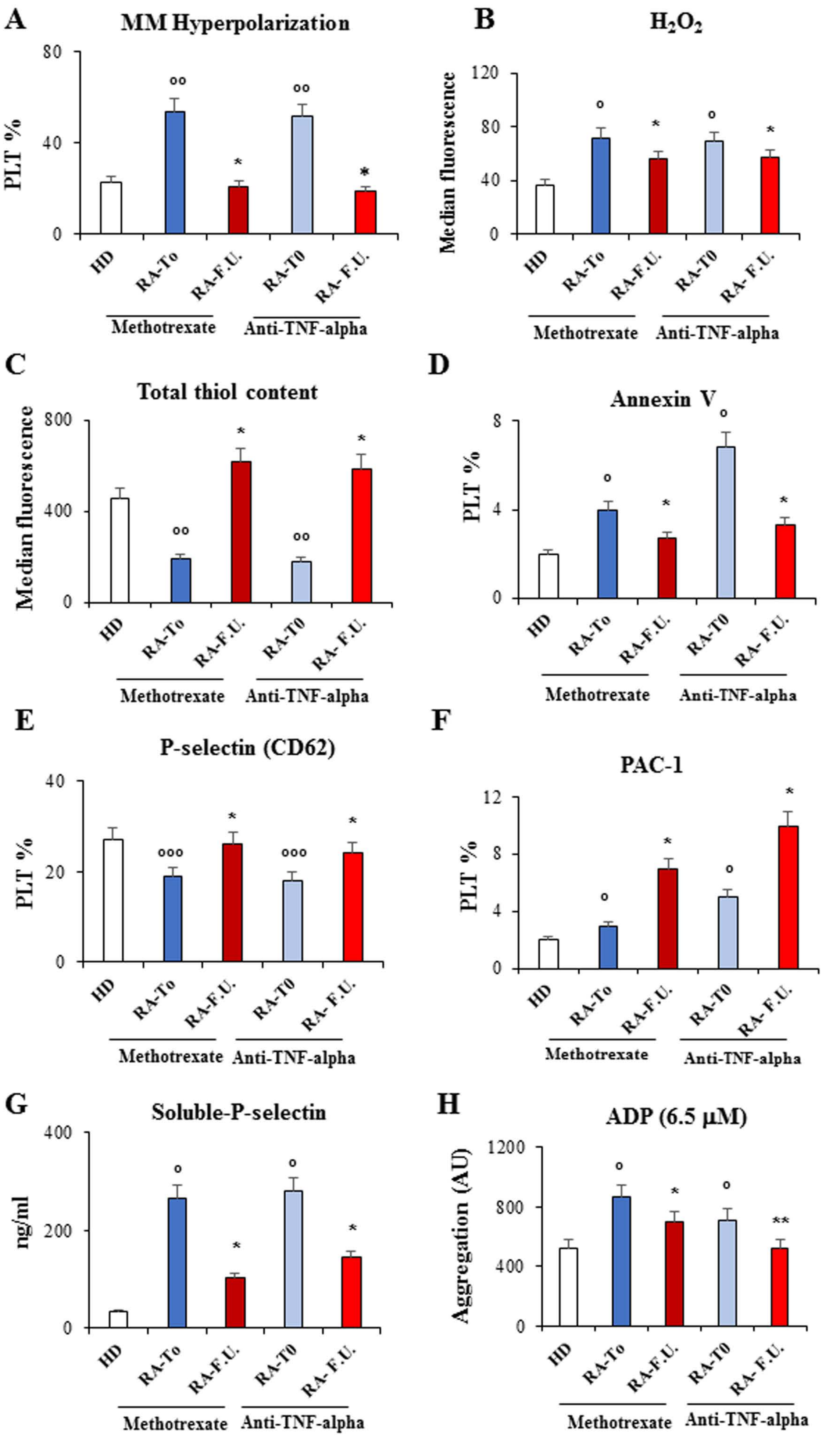

Supplementary Figure

Supplementary figure 1

Analysis of data from two rheumatoid arthritis (RA) patient cohorts: RA patients treated with Methotrexate and RA patients treated with anti-TNF-alfa.

Flow cytometry evaluation of:

(**A**) Percentage of platelets with hyperpolarized mitochondria membrane (MM). °°$p < 0.01$ (T0 *vs* HD); *$p < 0.05$ (F.U. *vs* T0);

(B) Levels of hydrogen peroxide (H_2O_2). °$p < 0.05$ (T0 *vs* HD); *$p< 0.05$ (F.U *vs* T0);

(**C**) Levels of total thiol content. °°$p<0.01$ (T0 *vs* HD); *$p < 0.05$ (F.U. *vs* T0);

(**D**) Percentage of platelet Annexine V positives. °$p < 0.05$ (T0 vs HD); *$p < 0.05$ (F.U. vs T0),

(**E**) Percentage of platelet P-selectin (CD62) positives. °°°$p < 0.001$ (To vs HD); *$p < 0.05$ (F.U. vs T0).

(**F**) Percentage of platelet PAC-1 positives. °$p < 0.05$ (T0 *vs* HD); *$p < 0.05$ (F.U. *vs* T0).

The numbers refer to mean ± SD of 45 women with RA, baseline (T0) and six months after DMARDs treatment (F.U.), and 25 age- and sex-matched healthy donors (HD).

For each patient, flow cytometry analysis was conducted in triplicate.

(**G**) Spectrophotometric analysis of soluble P-selectin in the plasma from HD and RA patients.

°$p < 0.05$ (T0 *vs* HD); *$p < 0.05$ (F.U. *vs* T0).

(H) Platelet aggregation evaluated in the whole blood from HD and RA patients (T0 and F.U.). The measurement was carried out after addition of 6.5 μMol ADP by an impedance multiplate aggregometer. The values are expressed in arbitrary aggregation units (AU). For each patient, platelet aggregation was conducted in triplicate. °$p< 0.05$ (T0 vs HD); *$p < 0.05$ (F.U. *vs* T0); **$p < 0.01$ (F.U. *vs* T0).
